# Pediatric Preventive Care in Middle-High Resource Countries—The Padova Chart for Health in Children

**DOI:** 10.3389/fped.2022.803323

**Published:** 2022-04-14

**Authors:** Alfonso Galderisi, Giorgio Perilongo, Sonia Caprio, Liviana Da Dalt, Giovanni Di Salvo, Michela Gatta, Carlo Giaquinto, Rosario Rizzuto, Adelaide Robb, Peter David Sly, Alessandra Simonelli, Annamaria Staiano, Roberto Vettor, Eugenio Baraldi

**Affiliations:** ^1^Department of Woman and Child's Health, University Hospital of Padova, Padova, Italy; ^2^Department of Pediatrics, Yale University, New Haven, CT, United States; ^3^Department of Biomedical Sciences, University of Padua, Padua, Italy; ^4^Division of Psychiatry and Behavioral Sciences, Children's National Hospital, Washington, DC, United States; ^5^Children's Health and Environment Program, Child Health Research Centre, The University of Queensland, Brisbane, QLD, Australia; ^6^Department of Developmental Psychology and Socialization, University of Padova, Padova, Italy; ^7^Department of Translational Medical Science, Section of Pediatrics, University Federico II, Naples, Italy; ^8^Department of Medicine, University Hospital of Padova, Padova, Italy

**Keywords:** pediatric health care, non-communicable chronic diseases, pediatric preventative care, lifestyle related disease, lifestyle and behavior

## Abstract

**Importance:**

The Padova Chart for Health in Children (PCHC) aims to gather the evidence of healthcare promotion and protection for chidren and adolescents (i.e., aged <18 y) into a single document in order to guide families, healthcare providers and social actors on healthy choices. No more than 2% of Europeans and North Americans aged <30 y have a healthy lifestyle. This, together with metabolic and brain plasticity during childhood, creates the ideal opportunity to implement preventive strategies. Guided interventions promoting healthy lifestyle in children and families therefore have a key role in abating the unprecedented pandemic of non-communicable diseases (NCDs) in adulthood.

**Observations:**

The PCHC is divided into four sections: nutrition, cardiovascular health, respiratory health, and mental and social health. Each section is structured in an ALICE approach (assessment, lobbying, intervention, call-for-action, evaluation): assessment of necessity, describing relevance to healthcare; lobbying to identify those who can effect the proposed interventions; interventions involving family, school and peers; a call-for-action to define priorities among the proposed interventions; and objective evaluation measures that can be applied on a population basis.

**Conclusions and Relevance:**

Interventions promoting health in childhood require joint action from multiple institutional, local and family representatives, with the shared goal of promoting health across the entire age group. These lifestyle interventions have the potential to change the lifetime risk trajectory for NCDs.

## Introduction

Optimizing pediatric health is key to reducing social inequality and ensuring sustainable growth. Although 30% of the European and North-American population is younger than 30 years (1), only 2% of this group has a healthy lifestyle, defined as being physically active for at least 60 min/d, consuming fruit and vegetables daily, spending <2 h/d on screen-based activities, and abstaining from alcohol and tobacco (2). This creates the conditions for an unprecedented pandemic of non-communicable diseases (NCDs), many of which start early in life, although not always with an overt onset in childhood or early adulthood (3). NCDs directly or indirectly account for ~90% of deaths in middle-to-high income countries (4). More recently, the combination of NCDs (specifically diabetes, obesity, hypertension, chronic lung and cardiovascular diseases, and mental illnesses) and socio-economic disparities have worsened outcomes in those affected by COVID-19, compelling national healthcare systems to target health as the only truly sustainable strategy to “prevent a “syndemic” disease” approach (5, 6). Targeting health promotion in children is ideal, as they have a metabolic (7, 8) and brain plasticity (9, 10) that offers the opportunity to instill life-long healthy habits (11). Interventions promoting healthy lifestyle in childhood are the sole sustainable and effective action to prevent NCDs in adulthood (1, 11).

We therefore decided to gather current evidence and guidance into a single document to inform and promote child health. We identified four areas, based on their potential impact on morbidity and mortality (4). 1. nutrition; 2. cardiovascular health; 3. respiratory health, 4. mental and social health.

## Methods

Each area was investigated by a team of two experts. Research was performed using PubMed, UpToDate, and WebOfScience. Guidelines and position statements published by European and North American scientific societies were also considered, as were position statements from the World Health Organization and Centers for Disease Control, systematic reviews, and Cochrane Reviews. The Chart targets “children” according to the broad UNICEF definition as those aged <18 years.

Each section is formatted using a call-for-action (ALICE) approach: **A**ssessment of necessity, based on current evidence; **L**obbying: identifying targets (families, primary healthcare providers and/or schools) to effect the proposed interventions; **I**nterventions and guidance involving family, school and peers; **C**all for action to define priorities among the proposed interventions; **E**valuate: proposed measurable population-level indicators to give a long-term assessment of the effectiveness of the interventions.

## Discussion/Observations

### Nutrition

#### Assessment of Necessity

Over the past 30 years, the prevalence of overweight and obesity have increased 10-fold in youth (12, 13), with 20% of those aged 2–19 years now obese (14), and one out of four having impaired glucose metabolism. Childhood obesity increases the risk of NCDs in adulthood, including type 2 diabetes, cardiovascular disease, asthma and other respiratory problems, sleep disorders, and liver disease (15). Further, longitudinal data from the SEARCH for Diabetes in Youth and the Restoring Insulin Secretion studies (16–18) have highlighted the morbidity in children with early type 2 (i.e., obesity-related) diabetes. This is important as more than 50% of those with childhood-onset type 2 diabetes have kidney disease or retinopathy by the age of 30 y (19). Treatment of childhood-onset type 2 diabetes focuses on dietary and behavioral interventions, with limited effective pharmacologic interventions available. Indeed, nutrition and lifestyle interventions remain the key tools to prevent childhood obesity and its comorbidities (20, 21). Ultra-processed food (22) and high-fructose beverages (23) are major sources of energy for children, both of which are associated with higher cardiometabolic risk (24–26). Despite this, children are exposed to the marketing of such products through techniques that exploit their developmental vulnerabilities.

It has been estimated that dietary changes may prevent ~11 millions deaths per year, that represent from the 19% to the 24% of deaths in adulthood. The benefit deriving from a healthy diet may be accounted as direct benefits—when directly impacting the disease determinants (e.g., reducing fat-saturated food directly impact cardiovascular risk) and indirect. These latter include the global impact of choosing sustainable foods, that include preferring plant-based dietary patterns (fruits, vegetables, nuts, seeds and whole grains—while limiting animal source foods), reducing food waste, and improving food production practices. Such an approach would imply a new definition of agricultural priorities and would imply an action at different levels of the food chain.

Sustainability of food results from the action of different “influencers”—determinants (27), that involves the food chain of production, the individual and collective choices and, ultimately, the food consumption. This latter is by itself a major determinant of food sustainability, thereby healthy food choices may drive food production chain in a bidirectional way.

#### Lobbying

Nutrition interventions should target not only children themselves, but their families and schools, along with food industries. Sustainable food promotion targets consumers but also regional and national authorities deputed to the agriculture to shift the current food-production paradigms.

#### Interventions

We identified two main intervention areas (20, 21): healthy food choices, education in food preparation. The access to age-appropriate high-quality nutrition is an important step toward combating malnutrition, one of the WHO millennium goals (28, 29). Malnutrition in Europe and North America is largely due to poor-quality nutrition (23). Food marketing, and children's own food preferences (including their requests for purchase and consumption) are major determinants of the obesity epidemic. Governments in France, Israel, and the United Kingdom have endorsed labeling policies that provide qualitative information on nutrient content, including highlighting excess salt and fat, and, in certain cases, age-appropriateness. Considering the needs of children when labeling food is a mandatory step if governments are to support healthy choices. Our recommendations combine guidelines from the American Heart Association (AHA) (30), WHO (31), and UNICEF (27), and target the two environments where food is consumed during childhood: schools and homes. As summarized in Table 1, interventions are meant to guide food choices, instead of defining age-adjusted dietary regimens. This strategy has been proven to be effective in large clinical studies (20, 21) and regional programs.

**Table 1:** Nutrition.

Assessment of necessity	• 20% of children aged 2–19 years are obese (14). • 25% of obese children have prediabetes (impaired fasting glucose, impaired glucose tolerance, or both) (32). • Childhood-onset type 2 diabetes is associated with microvascular complications before adulthood (19). • Lifestyle is main risk factor for prediabetes and diabetes (18, 33–35).
Interventions	• Healthy food choices (20, 21) • Consume vegetables and fruit daily. • Use vegetable oils and margarines that are low in saturated fat and trans fats, instead of butter or other animal fats. • Use wholegrain breads and cereals rather than refined grain products. • Consume skimmed or low-fat milk and dairy products daily. • Eat more fish, especially oily fish, broiled/grilled or baked. • Reduce salt intake, including in processed foods. • Reduce consumption of sugar-sweetened beverages and foods • Limiting animal source foods (27, 36) • Prefer products from a sustainable food chain (regional diversified vegetables and fruits) • Food preparation education (20, 21). • Promote and support breastfeeding.
Call for action	• Enforce sustainability and healthy choices in schools. • Mandate food “labeling-for-children” (23, 37, 38). • Introduce legislation supporting accessibility to healthy choices, including subsidies for low-income families (39).
Evaluation	• Track regional trends in obesity and glucose intolerance in children. • Introduce quality assessment of school-based nutrition.

Educational programs aimed to implement healthy food choice have a successful track record: family-based lifestyle intervention targeting for children and their families—and accounting for minorities and divesified groups—have been proven to successfully impact glucose tolerance and body weight in a safe and sustainable way (20, 21).

The ***Call for action*** includes proposals aimed at communities and industry.

The longitudinal **Evaluation** of the interventions will rely on regional studies tracking the prevalence of obesity and glucose metabolism impairment. Local authorities are expected to verify quality assessment of school nutrition.

### Cardiovascular Health

#### Assessment of Necessity

The AHA defines good cardiovascular health through seven health behaviors: abstinence from smoking; body-mass index <85th percentile; ≥60 min of moderate or vigorous physical activity daily; a diet emphasizing fruits, vegetables, fish, and whole grains, low in sodium and with few sugar-laden foods and drinks (40, 41); total cholesterol <170 mg/dL; blood pressure <90th percentile; and a fasting plasma glucose level <100 mg/dL (42). Less than 50% of adolescents aged 12–19 y achieve at least five out of these seven behaviors. As ~80% of cardiovascular events (CVD) could be prevented through these health behaviors (43), educational interventions starting in childhood are expected to have the highest impact on the incidence of CVD in adulthood (41). Beside nutrition (*see Section* Introduction), physical activity and screen time are the two main behavioral interventions able to affect the lifetime trajectory of cardiovascular risk.

#### Physical Activity

Physical activity (structured or unstructured) for preschool (44, 45) and school children (46) is a major determinant of pediatric health. Physical activity in preschool age is important to develop large motor skills and foster coordination (important aspects of school readiness), yet infants and preschool children in many middle- and high-income countries spend more than 30% of their time in sedentary activities such as screen time (45, 46).

*Screen time* is defined as the time spent engaging with visual screen-based technologies such as televisions, computers, videogames, smart phones, and tablets, including accessing the Internet and social media. In the last decade, there has been a widespread cultural adoption of media devices in young children; in one French study in 2018, 90% of those aged 2 y used touch-screen devices (47, 48). In this rapidly evolving digital age, much more time is spent in front of screens than previous generations; children's screen use is a key concern for parents and healthcare providers (49).

Evidence suggests that moderate screen time can be beneficial to children's wellbeing, widening social connections and improving learning skills, especially from activities including education and early learning. However, excessive use can be detrimental (50, 51), with potential negative impacts on physical, cognitive, emotional, and social wellbeing (48, 52), including a range of adverse physical, psychosocial, and cognitive outcomes (53). Further, the “time displacement hypothesis” suggests that excessive screen time displaces important protective health behaviors such as physical activity, green time, and adequate sleep (54). In addition, excessive screen time and social media use by children is associated with the development of obesity, sleep disturbances/problems (55), language delay, inattention (or attention disorders) (56), and depression (53, 57, 58). Yet, <20% of pediatricians ask families about their children's use of electronic devices (59, 60).

#### Lobbying

Proposed interventions target families, schools and primary healthcare providers.

***Interventions*** to promote physical activity and reducing screen time are summarized in Table 2. Age-specific recommendations are provided and should be explained with oral and written informative factsheets to families. Healthcare providers are the primary information source to effectively promote physical activity in preschool children, and one of the main sources of information in school-aged children along with schools. Universal screening for dyslipidemia at 9–11 years is still controversial (37), though scientific societies convened for the necessity for screening at risk children (overweight/obese children with/without a family history of early CVD) (Table 2) (61).

**Table 2:** Cardiovascular health.

Assessment of necessity	• >20% of adolescents have high blood pressure, high total cholesterol and low physical activity (41). • Most adolescents do not achieve at least five of the American Heart Association's seven health behaviors: not smoking; weight; active life; healthy diet; cholesterol; blood pressure; blood sugar.
Intervention 1 (physical activity)	Family and school: • Play with your children. • Encourage active toys (balls, jump ropes, outdoor toys). • Do not overschedule your children's day. • Discourage eating in front of a screen. • Avoid high caloric snacks and sugary drinks. • Do not use food as a reward after physical activity. Healthcare providers: • Enquire about physical activity habits of children and their parents/carergivers. • Describe the benefits to parents and families of an active lifestyle: Strengthen bones; decrease blood pressure; reduce stress and anxiety, and boost self-esteem; prevent obesity and type 2 diabetes; and prevent major cardiovascular events in adulthood. • Screen for dyslipidemia in all children at 9–11 year in overweight/obese children (61, 62). • Screen for dyslipidemia in children at 2 year in those with a family history of dyslipidemia or early cardiovascular disease • Screen for dysglycemia in overweight/obese children. • Check blood pressure annually. Age specific physical activity interventions: (<1 year): • >30 min of tummy time spread throughout the day. *3–5years:* • Active play. • Take account of the child's preferences. *6–17 years:* • ≥1 h moderate-to-vigorous physical activity every day. • ≥3 times per week preferred structured physical activity (football, running, gymnastics, group sports).
Intervention 2 (screen time)	Family • No screens during meals and for 1 h before bedtime. • Avoid having televisions and screen-based electronic devices in the children's bedroom. • Parents should be aware of positive and negative effects of screen time and monitor children's media content and the apps that are used or downloaded. Healthcare providers • Healthcare providers should regularly inquire about children's social media habits, and be familiar with the social media to which children may be exposed. • Parents and healthcare providers should ensure that sedentary screen time is not a routine part of child care. • Unregulated video streaming apps (e.g., YouTube and YouTube Kids) are not recommended in pre-school children. • Apps to control/limit screen time can be discussed and explored with parents. Age specific screen-time recommendations: <2 years • Avoid screen time in children younger than 2 y (except video chatting such as Skype and FaceTime when talking with relatives/family members). 2–5 y • Limit screen time to max 1 h/d. • Co-view with parents is recommended. 5–8 years • ≤2 h/d recreational screen time.
Call for action	• Provide accessible outdoor spaces for children. • Encourage school-based physical activity.
Evaluation	• Regional prevalence of childhood overweight/obesity.

The ***Call for action*** involves institutional local and regional groups to facilitate access to spaces for physical activity.

The ***Evaluation*** of the proposed interventions is through the prevalence in children of the seven cardiovascular health behaviors.

### Respiratory Health

#### Assessment of Necessity

Chronic respiratory diseases (CRD)—and in particular chronic obstructive pulmonary disease (COPD) and asthma—contribute significantly to the global burden of NCDs, and are major causes of morbidity and mortality. Tobacco smoking, e-cigarettes (vaping), and air pollution increase the burden of CRD. Further, although e-cigarettes have been proposed as an aid to smoking cessation in adults, there is mixed evidence for their effectiveness (63), and they can be a gateway to tobacco and nicotine use in adolescents naïve to tobacco (64, 65), E-cigarette users outnumber traditional smokers among adolescents (66); in one study 27.5% of high school and 10.5% of middle school students were current e-cigarette users (67), and in another, half of e-cigarette users aged 15–17 y had never used combustible cigarettes (68). This high use of e-cigarettes is of concern, as adolescents using e-cigarettes have an increased incidence of chronic bronchitis and asthma exacerbations compared with non-users (69, 70). E-cigarette use is also becoming more common in pregnant women, thus exposing the developing fetus to nicotine, which crosses the placenta and thus reaches the fetal bloodstream. Nicotine is not only a known teratogen (71, 72), but has been associated with major congenital anomalies such as cleft palette, prematurity and stillbirth (73, 74).

#### Pollution

Pollution is a major contributor to global morbidity and mortality (75). Children are especially vulnerable to the effects of pollution as they receive a relatively higher dose of toxins in any given environment, and are also more susceptible due to the physiology of their developing airways (76). Further, maternal exposure to pollutants during pregnancy may affect lung development in offspring (75). There is also convincing evidence that air pollution has negative impacts on respiratory health during childhood, and is associated with reduced maximal lung growth function (77, 78), potentially leading to the development of asthma (79) and COPD (80, 81).

Asthma is more common in children exposed to particulate air pollution, and there is evidence of a correlation between traffic-related air pollution and asthma occurrence (82, 83). Ambient air pollution is therefore recognized as a preventable risk factor for a spectrum of pediatric health problems (84).

#### Lobbying

Interventions should target families, healthcare practitioners and community/social organizations.

***Interventions*** include educational sessions to be delivered through healthcare providers and schools, and local-community programs and regional politics aimed to reduce pollution exposure. Interventions are summarized in Table 3.

**Table 3:** Respiratory health.

Assessment of necessity	• 27.5% of high school and 10.5% of middle school students use e-cigarettes (67). • E-cigarette users have an increased incidence of wheezing, chronic bronchitis and asthma exacerbations (69, 94, 95). • Air pollution is associated with reduced lung function (77, 78), and a higher prevalence of asthma (79) and COPD (80–82).
Interventions	Families: • Reduce exposure of infants and children to peak-daytime pollution. Healthcare providers: • Discourage e-cigarette and tobacco use. • Educate on the risk associated with e-cigarette and tobacco consumption. • Provide guidance on quitting. • Educate families on reading pollution forecasts, especially in metropolitan areas (75). Community: • Ban the sale of e-cigarettes and tobacco products, at least until the age of 18 years. • Regulate e-cigarette advertisement, as for tobacco-containing products. • Support taxes on nicotine-containing e-cigarettes (38). • Support public health-led education campaigns for schools and parents about the health risks of e-cigarettes (38).
Call for action	• Ban the sale of e-cigarettes to those <18 years (38). • Ban advertising of e-cigarettes.
Evaluation	• Prevalence of tobacco and e-cigarette use in those aged <18 years. • Prevalence of asthma in children and COPD in adulthood.

The ***Call for action*** targets e-cigarette regulatory policies, which are currently highly heterogeneous (85).

The efficacy of the proposed interventions can be *evaluated* through the prevalence of asthma in childhood and of COPD in adulthood from population studies.

### Mental and Social Health

#### Assessment of Necessity

Neuropsychiatric conditions are the leading cause of disability in young people, with half of all chronic mental illnesses beginning by the age of 14 years. Suicide is the second cause of death in adolescents and young adults, with 10 out of 100,000 adolescents completing suicide each year (86), while attention-deficit/hyperactivity disorder (ADHD), behavior problems, anxiety, and depression are the most common mental disorders in children (87). Further, gender discrimination and economic inequity are key determinants of mental health in children (62, 88, 89).

The COVID-19 pandemic, with the resulting quarantine, social isolation, mortality, and lack of proper education, has dramatically accelerated the course of a variety of childhood mental illnesses. Community includes family, schools, and peers; thus, to achieve optimal mental wellbeing a child needs support across these domains. Gender identity, self-image and family/school environment are the three key areas for childhood mental wellbeing.

Gender interacts with, but is different from, the binary categories of biological sex. Girls (but not boys) are more likely to view girls as victims of discrimination than boys, and children with egalitarian gender attitudes are more likely to perceive discrimination than are their peers (39, 90). Youths with lesbian, gay, bisexual, transgender, or queer (LGBTQ) orientations have higher rates of anxiety and mood disorders, as well as suicide and suicide attempts. Promoting acceptance of gender diversity allows children to develop without the burden of social isolation and discrimination that people may experience surrounding their gender identity.

The health impact of social media on children is greatest on mental health, and specifically self-esteem and wellbeing, with related issues around cyberbullying, with an association between the use of social media and self-esteem or *body image*. One of the more recent impacts of social media during the COVID-19 pandemic has been a sharp increase in eating disorders among children, in particular female teenagers. These vulnerable children restrict food and exercise excessively, presenting to pediatric wards with signs and symptoms of extreme starvation.

Income remains a major determinant of family and individual mental health (88). A population study in Great Britain found that the more debt people had, the more likely they were to have some form of mental disorder, even after adjustment for income and other sociodemographic variables. A review of European population surveys found that depression and anxiety are associated with low educational attainment, material disadvantage and unemployment, and for older people, social isolation. The pattern of social distribution of common mental disorders is observed as a social class gradient and is more marked in women than in men.

The impact of economical inequity goes beyond the paradigm of mental health: families' income impact the access to healthy food and food insecurity is, in turn, a major determinant of pediatric obesity. Thereby, the sociodemographic interactions– as resulting from housing policies, social interactions, neighborhood features—determine the individual risk of pediatric and adulthood obesity (62, 91).

The concept of ACEs—adverse childhood experiences—such as poverty, discrimination, loss of a parent, insecure housing put children at higher risk of poor physical and mental health outcomes in adulthood. Interventions to reduce and mitigate ACEs will have tremendous payoffs as these children grow into working happy and healthy adults.

Exposure to violence and poverty during childhood and adolescence is an independent risk factor for risky behaviors as substance abuse and alcohol use among adolescence. In spite of a transient decrease for substance abuse during the first year of COVID-19 pandemic, almost 5% of youth between 8 and 12th grade use illicit drugs other than marijuana and ~17% of eight graders consume alcohol (92).

#### Lobbying

Mental health cannot be improved by policies focused only on the most disadvantaged, but should consider the community as a whole, supporting children's capability to do and to be. Policies should be universal yet proportionate to need. Focusing solely on the most disadvantaged will fail to achieve the required reduction in health inequalities necessary to reduce the steepness of the social gradient in health. Families, schools, communities are the target bodies for interventions aimed to preserve and promote mental and social health of youth.

The ***interventions*** are based on the scientific consensus that giving every child the best possible start will generate the greatest societal and mental health benefits (93). Certain subgroups are at a higher risk of mental disorders because of greater exposure and vulnerability to unfavorable social, economic, and environmental circumstances, interrelated with gender.

Facilitating the access to diagnosis and support for children and families living with mental health disorders is of pivotal importance. Recommended interventions are deployed at schools (presence of advisors and supports), at home to support families within the domestic environment or to ensure a shelter environment as necessary, and in local communities (Table 4). Screening for substance abuse in primary care should be advised starting 8th grade (92).

**Table 4:** Mental and social health.

Assessment of necessity	• Neuropsychiatric conditions are the leading cause of disability in children (86). • 10:100,000 adolescents complete suicide each year (86). • 5% youth use illicit drugs other than marijuana (92).
Interventions	School: • School-advisor support (psychologist). • Favoring commitment and engagement over competitive behaviors (such as school marks) through complimentary educational rewards. • Gender-discrimination teaching programs (39). Families: • Parenting to parenting. • Community-based shelter programs. • Community-based family programs. • Supporting parents, especially single parents, in learning new job skills, so they can remain employed and continuing to provide childcare while working. • Specialized childcare when children are ill, so parents do not need to miss work to provide care. Primary Care: • Screening for substance abuse starting 8th grade • Discussing income support program accessibility Communities: • Sustainable daycare for younger children, and year-round school for older children. • Local campaigns targeting family and maternal income-support (fight family poverty) • Public campaign for gender-equality (public reports on gender discrimination, advertisements, fashion, online restrictions) (62, 88, 89). • Addiction-focused educational campaigns (gaming, tobacco, e-cigarettes, social media, alcohol, and other drugs). • Financial support of educational activities to minimize social inequality, providing additional support as youths transition through elementary school to high school, college and post-graduate education. • Provide academic support for students in college who come from disadvantaged backgrounds.
Call for action	• Family and mother-oriented income support policies • Gender non-discriminative policies at school and on social media. • Engaging, non-competitive school environment. • Shelter houses for children and mothers. • Tutoring program so that youths struggling academically can reach out to other students for extra help.
Evaluation	• Develop a quality certification process for public and private schools. • Loss of school days.

The ***call for action*** to maximize the impact of the proposed interventions involves supporting shelter houses for children and mothers, creating engaging, non-competitive school environments, and gender non-discriminative policies at school and in the media. These interventions are expected to reduce the risk determinants of mental health disorders in children, though their implementation relies on regional and state policies.

Community-based interventions targeting poverty remain a major leverage to promote mental health throughout all the age groups.

The ***evaluation*** of the efficacy of regional and state interventions could be quantified through certification processes for schools and educational environments. Additionally, regional school performance could be an indirect measure of effective mental health programs on a regional basis.

## Conclusions

Less than 2% of young adults (<30 years) have a healthy lifestyle, and no more than 50% of adolescents aged 12–19 years meet at least five of the seven ideal cardiovascular health behaviors. Childhood presents a unique window of opportunity to effect strategic interventions to promote healthy lifestyles, due to the metabolic and brain plasticity of children. Interventions targeting childhood therefore hold the potential to dramatically abate the rising incidence of NCDs and should involve families, schools and community-social groups. The ALICE approach we propose aims to define priority areas, targets and interventions, actions and measurable indicators to be used by primary healthcare providers and pediatricians to address health promotion strategies in childhood.

## Author Contributions

EB and AG designed the project and drafted the manuscript. GP, MG, ASi, and AR contributed to the Mental and Social Health section. AG and SC contributed to the Nutrition section. GD and RV contributed to the Cardiovascular Health section. EB and PS contributed to the Respiratory Health section. LD, CG, ASt, RR, and EB critically revised the manuscript. All authors approved the final version of the document.

## Funding

This document was supported by an unrestricted grant from the University of Padova on the occasion of the 800 years anniversary (800 years Committee 1222-2022).

## Conflict of Interest

The authors declare that the research was conducted in the absence of any commercial or financial relationships that could be construed as a potential conflict of interest.

## Publisher's Note

All claims expressed in this article are solely those of the authors and do not necessarily represent those of their affiliated organizations, or those of the publisher, the editors and the reviewers. Any product that may be evaluated in this article, or claim that may be made by its manufacturer, is not guaranteed or endorsed by the publisher.
